# Differential effects of foot-and-mouth disease infection and vaccination on fertility of Holstein–Friesian heifers: Evidence from a within-animal retrospective study in Indonesia

**DOI:** 10.14202/vetworld.2026.3176-3190

**Published:** 2026-07-20

**Authors:** Habib Asshidiq Syah, Widi Nugroho, Aulia Puspita Anugra Yekti, Nurul Isnaini, Sri Wahjuningsih, Mashudi Mashudi, Tri Eko Susilorini, Suyadi Suyadi, Muhaimin Rifa'i, Putri Utami, Anggita Dian Pramudhita, Korawan Sringarm, Trinil Susilawati

**Affiliations:** 1 Faculty of Animal Science and Technology, Universitas Brawijaya, Malang, East Java, Indonesia.; 2 Faculty of Veterinary Medicine, Universitas Brawijaya, Malang, East Java, Indonesia.; 3 Faculty of Science, Technology, and Mathematics, Universitas Brawijaya, Malang, East Java, Indonesia.; 4 Faculty of Agriculture, Chiang Mai University, Chiang Mai, Thailand.

**Keywords:** artificial insemination, fertility, foot-and-mouth disease, Holstein–Friesian heifer, Indonesia, reproductive performance, vaccination, within-animal study

## Abstract

**Background and Aim:**

Foot-and-mouth disease (FMD) remains one of the most economically important transboundary diseases affecting livestock worldwide. Following its re-emergence in Indonesia in 2022 after more than three decades of freedom, concerns arose regarding its impact on dairy cattle fertility and the possible reproductive consequences of emergency vaccination. However, field-based evidence directly comparing the reproductive effects of natural FMD infection and vaccination within the same animals is lacking. This study evaluated the association of clinical FMD infection and inactivated FMD vaccination with reproductive performance in Holstein–Friesian heifers using a within-animal retrospective design.

**Materials and Methods:**

A retrospective within-animal before-and-after study was conducted using reproductive records from 772 Holstein–Friesian heifers maintained by 501 smallholder dairy farmers in Malang Regency, East Java, Indonesia. Reproductive performance during two consecutive 180-day periods before-and-after FMD infection or vaccination was compared within the same animals. Heifers were classified into three conditions: clinical FMD during the outbreak (n = 464), clinical FMD after the first vaccination (n = 60), and vaccinated heifers remaining clinically healthy (n = 248). First-service conception rate (FSCR) and pregnancy rate (PR) were analyzed using mixed-effects logistic regression, whereas first-service-to-conception interval (FSCI) and reproductive record exit were evaluated using Kaplan–Meier and Cox proportional hazards models.

**Results:**

Clinical FMD infection significantly impaired reproductive performance. In condition 1, FSCR and PR decreased significantly after infection (FSCR: odds ratio [OR] = 0.49, 95% confidence interval [CI]: 0.35–0.70, p < 0.01; PR: OR = 0.43, 95% CI: 0.33–0.57, p < 0.01). The hazard of conception was also significantly reduced (hazard ratio = 0.56, 95% CI: 0.46–0.67, p < 0.01), with the median FSCI increasing from 27.5 to 45 days. Vaccinated heifers that subsequently developed clinical FMD showed no significant deterioration in reproductive performance. Vaccinated clinically healthy heifers exhibited only a modest reduction in PR (OR = 0.69, 95% CI: 0.48–0.99, p = 0.04), while FSCR remained unchanged. Exploratory analysis indicated that artificial insemination performed >90 days after infection or vaccination was associated with improved conception outcomes.

**Conclusion:**

Natural FMD infection substantially reduced fertility in Holstein–Friesian heifers, whereas vaccination produced comparatively minor and less consistent reproductive effects. These findings support continued FMD vaccination programs while highlighting the importance of integrating vaccination schedules with reproductive management to minimize fertility losses during FMD outbreaks.

## INTRODUCTION

Foot-and-mouth disease (FMD) is a highly contagious viral disease affecting cloven-hoofed livestock, including cattle, sheep, goats, and pigs [1]. It is caused by FMD virus (FMDV), a member of the genus *Aphthovirus* within the family *Picornaviridae* [2]. FMD adversely affects the productivity and profitability of livestock production systems by reducing animal performance and causing substantial economic losses. In dairy cattle, FMD has been associated with impaired reproductive performance through multiple physiological mechanisms. Affected animals frequently experience reduced feed intake because of painful oral lesions, resulting in negative energy balance and disruption of reproductive endocrine function [3, 4]. Infection occurring during the breeding period may impair fertilization and early embryonic development up to the morula stage [5]. Furthermore, FMD has been associated with increased age at first calving [6], higher embryonic mortality [7], an increased incidence of reproductive disorders [8], elevated services per conception (S/C), prolonged days open, and extended calving intervals [9, 10]. These adverse reproductive consequences may prevent heifers from achieving the fertility performance commonly reported for Holstein–Friesian cattle under tropical production systems, including first-service conception rate (FSCR) of approximately 24%–47.3% [11, 12], pregnancy rate (PR) of 57.77%–65% [13, 14], and S/C ranging from 1.98 to 2.53 [14, 15]. In addition, FMD infection may increase the risk of culling among surviving dairy cattle because of subsequent infertility and reduced reproductive efficiency [16].

After more than three decades of disease-free status, FMD re-emerged in Indonesia in May 2022, with confirmed cases rapidly spreading across several regions, including Malang Regency, East Java [17]. Molecular characterization demonstrated that the causative FMDV belonged to the O/ME-SA/Ind-2001e lineage [18], which was first detected in Myanmar in 2017 before subsequently spread to Thailand, Vietnam, Malaysia, and, subsequently, Indonesia [19]. The 2022 outbreak caused severe economic losses in the Indonesian dairy sector. Smallholder dairy farmers experienced substantial reductions in milk production, increased calf mortality, and considerable financial losses, estimated at approximately US$2,500 per farmer in herds averaging 6.8 lactating cows [20]. Milk production in Holstein–Friesian (HF) cows reportedly declined by up to 48% following FMD infection [21], while calf mortality reached 17.8% [20]. Despite these documented production losses, information regarding the reproductive consequences of FMD in Indonesian dairy cattle remains limited.

Vaccination remains one of the principal strategies for controlling FMD, alongside strict biosecurity measures and the continuous development of effective multivalent vaccines [22–24]. Nevertheless, concerns have been raised regarding the potential reproductive effects of FMD vaccination in dairy cattle. Previous studies have reported transient adverse effects following vaccination, including reduced feed intake and increased body temperature [25–27]. Such temporary physiological disturbances may compromise oocyte quality, alter progesterone secretion, and increase the risk of early embryonic loss [28, 29]. During the nationwide vaccination campaign following the 2022 outbreak, anecdotal observations from Indonesian dairy farmers and veterinarians suggested increased culling and reduced reproductive performance after vaccination. However, robust scientific evidence distinguishing the reproductive effects of vaccination from those of natural FMD infection under field conditions remains scarce.

Previous investigations evaluating the reproductive consequences of FMD infection or vaccination have largely relied on comparisons between independent groups of animals. Although informative, such study designs are susceptible to between-animal variability arising from genetic background, management practices, nutritional status, environmental conditions, and individual reproductive potential. Consequently, the independent effects of natural FMD infection and vaccination on fertility remain difficult to distinguish. To date, no published study has used a within-animal before-and-after design to evaluate reproductive performance in HF heifers maintained under tropical smallholder dairy production systems before-and-after FMD infection or vaccination. Such a design allows each animal to serve as its own control, thereby minimizing individual-level confounding and improving the precision of treatment-effect estimation [30]. Moreover, no field study has directly quantified and compared the magnitude of reproductive changes associated with natural clinical FMD infection and vaccination within the same study population during the Indonesian FMD outbreak [31]. Addressing these knowledge gaps is essential for developing evidence-based reproductive management strategies and optimizing vaccination programs in FMD-endemic developing countries.

Therefore, this study aimed to evaluate within-animal changes in reproductive performance before-and-after three different exposure conditions: (i) clinical FMD during the outbreak, (ii) clinical FMD following vaccination, and (iii) vaccination without subsequent clinical FMD. The study further aimed to compare the magnitude of reproductive effects associated with natural FMD infection and vaccination using paired reproductive records from the same animals. We hypothesized that natural clinical FMD infection would result in greater impairment of reproductive performance than vaccination and that any reproductive effects associated with vaccination would be comparatively smaller and transient. The findings are expected to provide robust field-based evidence to support reproductive management strategies, optimize vaccination scheduling, and guide FMD control policies in dairy production systems within FMD-endemic developing countries.

## MATERIALS AND METHODS

### Ethical approval

This study was based exclusively on retrospective reproductive and herd health records routinely collected between 2021 and 2023 from a dairy cooperative in Malang Regency, East Java, Indonesia. No experimental interventions, invasive procedures, direct animal handling, biological sample collection, or manipulation of animals were performed specifically for research purposes. All data were generated during routine veterinary and reproductive management practices. Before analysis, the dataset was anonymized to remove all farmer and animal identifiers, thereby ensuring confidentiality and data privacy. In accordance with institutional and national guidelines governing research based solely on retrospective secondary records without direct animal involvement, formal approval from an animal ethics committee was not required.

### Study period and location

The study was conducted using reproductive records collected from a dairy cooperative comprising smallholder dairy farms in Malang Regency, East Java, Indonesia. The study period covered January 2020 to August 2023, encompassing both pre-outbreak and post-outbreak reproductive records. The FMD outbreak occurred between May 24 and August 8, 2022. Initial outbreak confirmation was performed by the Veterinary Diagnostic Laboratory (Wates, Yogyakarta, Indonesia), the national reference laboratory for FMD diagnosis. Because laboratory confirmation was not feasible for every affected animal during the outbreak, subsequent cases were diagnosed clinically by trained local veterinarians based on compatible clinical signs, including oral ulcers, hypersalivation, pyrexia, anorexia, lameness, and ulcerative lesions affecting the extremities.

Each animal maintained within the cooperative possessed an individual identification record linked to its ear tag, enabling complete documentation of artificial insemination (AI) services, reproductive status, pregnancy diagnosis, vaccination history, calving records, and the onset of clinical FMD.

During the national vaccination campaign, clinically healthy cattle received a primary dose of the inactivated FMD vaccine Aphtovaks-E™ (PT Vaksindo Satwa Nusantara, Bogor, Indonesia) in June 2022, followed by a booster dose of Avtogen™ (Biogénesis Bagó, Garín, Buenos Aires, Argentina) in July 2022. The heifers included in this study had no previous history of FMD vaccination; therefore, the June 2022 vaccination represented their first exposure to FMD immunization.

### Study design

This retrospective, within-animal, before-and-after observational study evaluated reproductive performance before-and-after natural FMD infection or vaccination, using paired reproductive records from the same animals. The within-animal design enabled each heifer to serve as its own control, thereby minimizing between-animal variability associated with genetics, management, and environmental factors.

Reproductive performance was compared during two consecutive observation periods before-and-after FMD exposure. The same heifer contributed data to both observation periods, allowing direct within-animal comparison of fertility outcomes following natural infection or vaccination.

### Study population

A total of 772 HF heifers from 501 smallholder dairy farmers were included in the study. All heifers received their first AI between 15 and 18 months of age.

For post-exposure evaluation, animals were classified into three mutually exclusive exposure groups according to their clinical status and the timing of FMD relative to vaccination:

1. Heifers that developed clinical FMD during the outbreak (Condition 1; n = 464).

2. Heifers that remained clinically healthy during the outbreak but subsequently developed clinical FMD after the first vaccination round (Condition 2; n = 60).

3. Heifers that remained clinically healthy throughout the outbreak and after the first vaccination round (Condition 3; n = 248).

**Figure 1:**
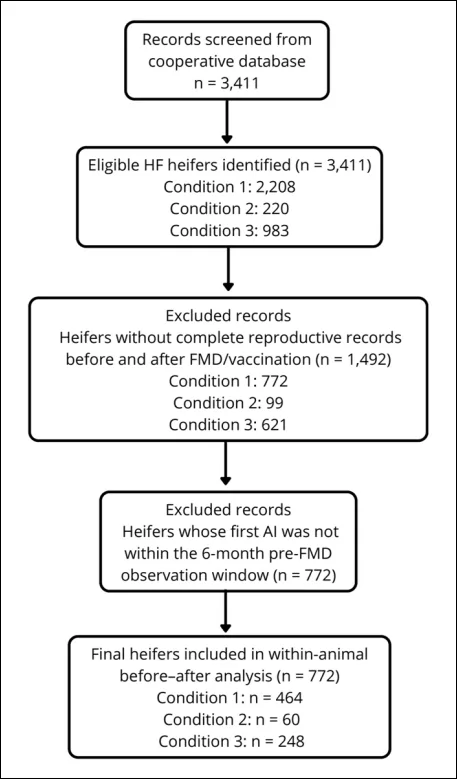
STROBE flow diagram showing the selection of Holstein–Friesian heifers included in the within-animal before-and-after analysis.

**Figure 2:**
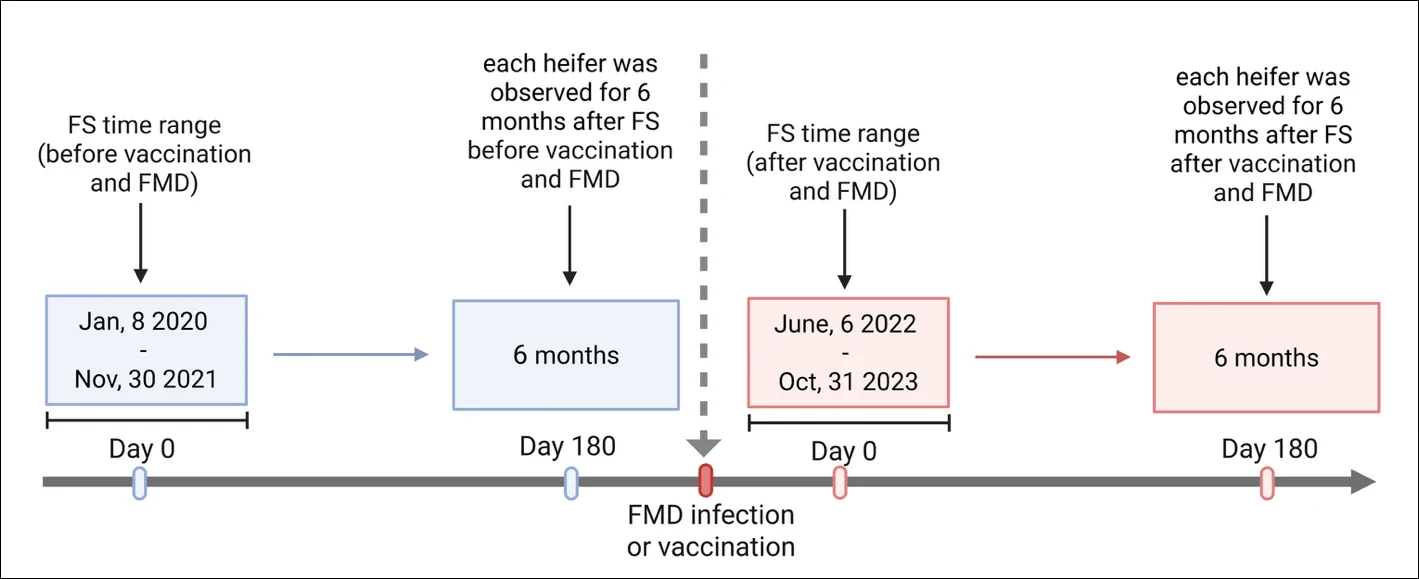
Schematic timeline illustrating the observation periods for reproductive performance in Holstein-Friesian heifers before-and-after the foot-and-mouth disease (FMD) outbreak in Indonesia. The pre-FMD observation period included heifers first inseminated between January 8, 2020, and November 30, 2021, whereas the post-FMD observation period included those first inseminated between June 6, 2022, and October 31, 2022. Each observation period lasted 180 days from the first service (FS), defined as the first artificial insemination.

The selection process for the study animals is illustrated in Figure 1, whereas baseline characteristics by exposure condition are presented in Table 1. All 772 heifers contributed paired pre-exposure and post-exposure reproductive records; therefore, no single-period records were included in the final analyses. The study timeline is presented in Figure 2.

**Table 1:** Baseline characteristics of Holstein–Friesian heifers according to exposure condition.

**Characteristics**	**Condition 1**	**Condition 2**	**Condition 3**
Exposure definition	Clinical FMD during the outbreak	Clinically healthy during the outbreak but developed clinical FMD after the first vaccination	Clinically healthy during the outbreak and remained free of clinical FMD after the first vaccination
Number of heifers, n	464	60	248
Number of smallholder farmers, n	362	50	204
Pre-exposure first AI period	January 8, 2020–November 30, 2021	January 8, 2020–November 30, 2021	January 8, 2020–November 30, 2021
Post-exposure first AI period	June 6, 2022–August 11, 2023	June 6, 2022–August 11, 2023	June 6, 2022–August 11, 2023

FMD = Foot-and-mouth disease; AI = Artificial insemination. Condition 1 = Heifers clinically affected by FMD during the outbreak; Condition 2 = Heifers that developed clinical FMD after vaccination; Condition 3 = Clinically healthy heifers that remained free of clinical FMD after vaccination.

### Eligibility criteria

HF heifers were eligible for inclusion if they:

1. were registered in the cooperative database;

2. possessed identifiable individual records linked to ear tag information;

3. had complete AI and pregnancy diagnosis records during both pre-exposure and post-exposure observation periods;

4. received their first AI between 15 and 18 months of age; and

5. could be assigned to one of the three predefined exposure conditions.

Heifers were excluded if they lacked complete reproductive records before-and-after FMD infection or vaccination, if the first AI did not occur within the predefined 6-month pre-FMD observation period, or if essential information regarding AI date, pregnancy diagnosis, FMD status, or vaccination history was incomplete. The selection process and reasons for exclusion are presented in Figure 1.

### Observation window and time origin

Reproductive performance was evaluated during two 180-day observation periods. During the pre-outbreak period, heifers receiving their first AI between January 8, 2020, and November 30, 2021, were followed for 180 days from the date of first insemination.

During the post-exposure period, follow-up commenced from the first AI performed after the relevant exposure date, defined as the date of clinical FMD onset for clinically affected heifers or the vaccination date for vaccinated clinically healthy heifers. The first AI initiating post-exposure follow-up occurred between June 6, 2022, and August 11, 2023. All inseminations occurring within the same 180-day period were considered part of the corresponding observation window.

### Outcome definitions

The primary reproductive outcomes were defined *a priori*. FSCR was defined as conception following the first AI within a 180-day observation period (yes/no) [32].

PR was defined as achievement of at least one confirmed pregnancy during the same 180-day observation period (yes/no) [14, 33].

S/C was defined as the number of inseminations required to achieve one confirmed pregnancy among heifers that conceived during follow-up [34].

First-service-to-conception interval (FSCI) was defined as the number of days from the first AI to the insemination resulting in confirmed pregnancy. Heifers conceiving at first-service were assigned an FSCI of 0 days.

Pregnancy diagnosis was performed approximately 60 days after AI by trained veterinary personnel using rectal palpation. A confirmed pregnancy was recorded following a positive rectal palpation result in the cooperative reproductive database. Embryonic or fetal loss was inferred when a heifer with a confirmed pregnancy failed to produce a subsequent calving record.

### Proxy definition of reproductive record exit

Complete records describing culling, sale, transfer, or mortality were unavailable; therefore, reproductive record exit was evaluated using a predefined proxy outcome.

A heifer was considered to have experienced reproductive record exit if, following the exposure date, she had no additional AI records during the 180-day follow-up period and no confirmed pregnancy record. The date of the final recorded AI was considered the proxy for the reproductive record exit date.

This definition was intended solely to identify the discontinuation of reproductive follow-up and should not be interpreted as confirmed culling due to infertility. Rather, it represents a pragmatic outcome derived from routinely collected AI records in smallholder dairy production systems where complete culling records are unavailable.

### Statistical analysis

FSCR and PR were analyzed using mixed-effects logistic regression models. Observation period (before versus after FMD), exposure condition, and their interaction were included as fixed effects, whereas heifer identity was incorporated as a random intercept to account for repeated measurements. Farm-level clustering was evaluated as an additional random effect and retained when supported by model fit. Results are presented as odds ratios (OR) with 95% confidence intervals (CI).

An exploratory post-exposure analysis evaluated FSCR according to the interval between clinical FMD onset or vaccination and the first post-exposure AI. Intervals were categorized as 0–30, 31–60, 61–90, and >90 days, with the >90-day interval serving as the reference category. Pairwise ORs were calculated within each exposure condition, and Fisher's exact test was used to compare each interval with the reference category. When zero conception events occurred, the Haldane–Anscombe correction was applied. Because of sparse observations, particularly in Condition 2, the results of this exploratory analysis were interpreted with caution.

S/C was summarized descriptively among heifers that conceived during follow-up, and no formal statistical comparisons were performed.

FSCI was analyzed as a time-to-event outcome using Kaplan–Meier survival analysis and Cox proportional hazards regression. Time was defined as the interval from first AI to confirmed conception, whereas non-pregnant heifers were right-censored at 180 days. For computational purposes, heifers conceiving at first-service were assigned an FSCI of 1 day. Cox models included the observation period, exposure condition, and their interaction, with robust standard errors clustered at the heifer level. Results are presented as hazard ratios (HR) with 95% CI.

Reproductive record exit was evaluated only during the post-FMD period using Kaplan–Meier survival analysis and Cox proportional hazards regression. Because the proportional hazards assumption was violated according to Schoenfeld residuals, a piecewise Cox proportional hazards model was fitted by dividing follow-up into 0–90-day and 91–180-day intervals and incorporating condition-by-time interactions.

All statistical analyses were two-sided, and statistical significance was defined as p < 0.05. Analyses were performed using R version 4.4.1 with the lme4**, **survival**, **dplyr**, **tidyr**, **ggplot2, and survminer packages (R Foundation for Statistical Computing, Vienna, Austria).

## RESULTS

## FSCR

The success of AI was confirmed by pregnancy diagnosis on day 60 post-insemination. The mixed-effects logistic regression model evaluating the association of FMD infection and FMD vaccination with FSCR is presented in Table 2. In Condition 1, FSCR decreased significantly after FMD infection compared with the pre-outbreak period (OR = 0.49; 95% CI: 0.35–0.70; p < 0.01). In contrast, no significant changes in FSCR were observed in Condition 2 (OR = 1.00; 95% CI: 0.39–2.54; p = 1.00) or Condition 3 (OR = 0.72; 95% CI: 0.45–1.14; p = 0.16).

**Table 2:** Mixed-effects logistic regression analysis of FSCR in Holstein–Friesian heifers before-and-after FMD infection or vaccination.

**Condition**	**Before,** **n pregnant/N**	**Estimated probability (95% CI)**	**After, n pregnant/N**	**Estimated probability (95% CI)**	**OR (After vs Before)**	**95% CI**	**p-value**
1	105/464	0.22 (0.18–0.27)	59/464	0.12 (0.09–0.16)	0.49	0.35–0.70	<0.01
2	11/60	0.18 (0.10–0.30)	11/60	0.18 (0.10–0.30)	1.00	0.39–2.54	1.00
3	51/248	0.20 (0.15–0.26)	39/248	0.15 (0.11–0.21)	0.72	0.45–1.14	0.16

FSCR = First-service conception rate; OR = Odds ratio; CI = Confidence interval. The before period was used as the reference category for OR estimation within each condition. OR <1 indicates reduced odds of conception in the after period. Condition 1 = Heifers clinically affected by FMD during the outbreak; Condition 2 = Heifers developing clinical FMD after vaccination; Condition 3 = Clinically healthy heifers after vaccination.

Table 3 presents the exploratory post-exposure analysis based on the interval from clinical FMD onset or vaccination to the first post-exposure AI. In Condition 1, first AI within 90 days after clinical FMD onset was associated with substantially lower odds of first-service conception than AI performed after >90 days, with reductions ranging from approximately 84% to 91%. In Condition 2, a similar numerical trend was observed, with the odds of first-service conception within 90 days being approximately 74%–78% lower than those after >90 days; however, these differences were not statistically significant, likely because of the limited sample size. In Condition 3, first AI within 90 days after vaccination was also associated with lower odds of first-service conception, with reductions ranging from approximately 60% to 73% compared with AI performed after >90 days.

### PR and S/C

Table 4 summarizes pregnancy outcomes and ORs before-and-after FMD infection or vaccination using mixed-effects logistic regression. In Condition 1, which included 464 heifers, the model-based estimated probability of pregnancy decreased from 0.57 before FMD infection to 0.37 after infection. The odds of conception after infection were significantly lower than those before infection (OR = 0.43; 95% CI: 0.33–0.57; p < 0.01). In Condition 2, which included 60 susceptible heifers that received FMD vaccination and subsequently developed clinical FMD, PRs were similar before-and-after vaccination. The model-based estimated probability of pregnancy was 0.45 before and 0.39 after vaccination, with no significant difference in PR (OR = 0.81; 95% CI: 0.38–1.69; p = 0.57). In Condition 3, which included 248 heifers, the model-based estimated probability of pregnancy decreased from 0.54 before vaccination to 0.45 after vaccination, and the odds of conception after vaccination were significantly lower than those before vaccination (OR = 0.69; 95% CI: 0.48–0.99; p = 0.04).

To our knowledge, this study provides the first quantitative field evidence showing that vaccination without subsequent clinical FMD was associated with only a modest 31% reduction in PR odds, compared with a 57% reduction after natural clinical FMD infection. Descriptively, S/C was slightly higher during the post-FMD period in Conditions 1 and 3, increasing from 2.19 ± 1.33 to 2.42 ± 1.39 and from 2.08 ± 1.12 to 2.38 ± 1.44, respectively. In contrast, little change was observed in Condition 2 (1.96 ± 1.04 vs. 2.00 ± 1.35).

**Table 3:** FSCR according to the interval from FMD onset or vaccination to first post-exposure AI in Holstein–Friesian heifers.

**Condition**	**Interval from FMD onset/** **vaccination to first post-exposure AI**	**Pregnant/Total**	**FSCR (%)**	**OR vs >90 days**	**95% CI**	**p-value**
1	0–30 days	0/11	0.00	0.09	0.01–1.59	0.02
	31–60 days	3/57	5.26	0.12	0.04–0.39	<0.01
	61–90 days	7/98	7.14	0.16	0.07–0.37	<0.01
	>90 days	95/298	31.88	Reference	Reference	Reference
2	0–30 days	0/4	0.00	0.26	0.01–5.38	0.55
	31–60 days	1/13	7.69	0.20	0.02–1.81	0.24
	61–90 days	1/12	8.33	0.22	0.02–1.98	0.24
	>90 days	9/31	29.03	Reference	Reference	Reference
3	0–30 days	3/30	10.00	0.27	0.08–0.96	0.03
	31–60 days	7/50	14.00	0.40	0.16–0.98	0.05
	61–90 days	5/43	11.63	0.32	0.12–0.89	0.02
	>90 days	36/125	28.80	Reference	Reference	Reference

FSCR = First-service conception rate; FMD = Foot-and-mouth disease; AI = Artificial insemination; OR = Odds ratio; CI = Confidence interval. Condition 1 = Heifers clinically affected by FMD during the outbreak; Condition 2 = Heifers that developed clinical FMD after vaccination; Condition 3 = Clinically healthy heifers after vaccination. The interval was calculated from the date of clinical FMD onset or vaccination to the date of the first post-exposure AI. Within each condition, the >90-day interval was used as the reference category. Pairwise ORs were calculated by comparing each interval category with the >90-day reference interval. The Haldane–Anscombe correction was applied to the OR estimate when zero conception events were present. Pairwise p-values were obtained using Fisher’s exact test. This interval-based analysis was considered exploratory because some strata had small sample sizes, particularly Condition 2.

**Table 4:** Mixed-effects logistic regression analysis of PR in Holstein–Friesian heifers before-and-after FMD infection or vaccination.

**Condition**	**Before, n pregnant/N**	**Estimated probability (95% CI)**	**After, n pregnant/N**	**Estimated probability (95% CI)**	**OR (After vs Before)**	**95% CI**	**p-value**	**S/C before**	**S/C after**
1	265/464	0.57 (0.53–0.62)	174/464	0.37 (0.33–0.42)	0.43	0.33–0.57	<0.01	2.19 ± 1.33	2.42 ± 1.39
2	27/60	0.45 (0.32–0.58)	24/60	0.39 (0.28–0.53)	0.81	0.38–1.69	0.57	1.96 ± 1.04	2.00 ± 1.35
3	133/248	0.54 (0.47–0.60)	111/248	0.45 (0.38–0.51)	0.69	0.48–0.99	0.04	2.08 ± 1.12	2.38 ± 1.44

PR = Pregnancy rate; OR = Odds ratio; CI = Confidence interval; S/C = Services per conception. The before period was used as the reference category for OR estimation within each condition. OR <1 indicates reduced odds of pregnancy in the after period. Condition 1 = Heifers clinically affected by FMD during the outbreak; Condition 2 = Heifers developing clinical FMD after vaccination; Condition 3 = Clinically healthy heifers after vaccination.

## FSCI

**Figure 3:**
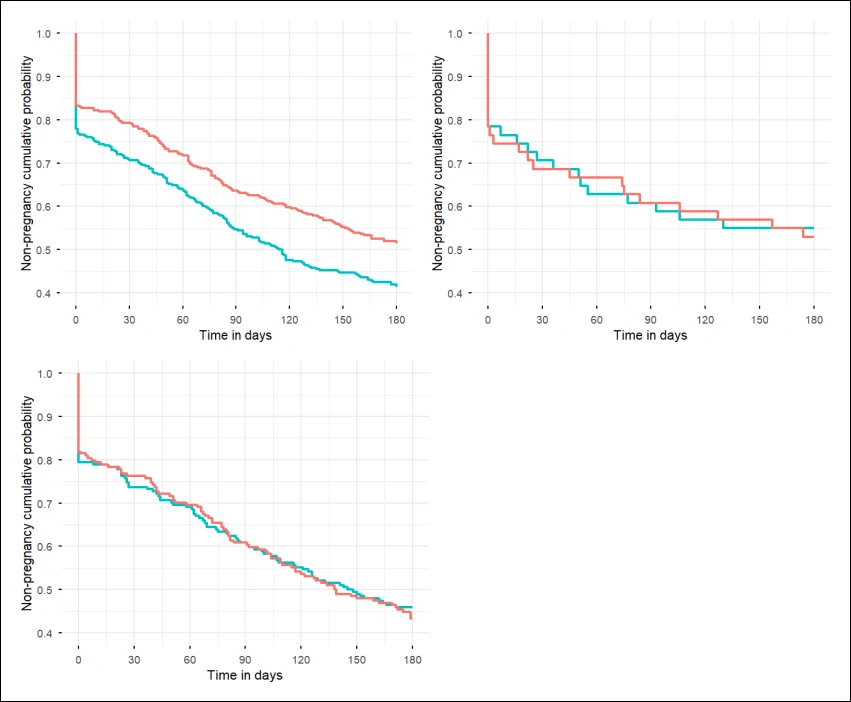
Unadjusted Kaplan–Meier survival curves for the interval from first AI to conception up to 180 days in HF heifers across three study conditions. Only heifers that remained in the herd and were eligible for reproductive follow-up were included in the analysis. Heifers that did not conceive by the end of the observation period were right-censored. The x-axis represents the number of days required to achieve conception, whereas the y-axis represents the cumulative probability of animals remaining non-pregnant during the observation period. (A) Condition 1; (B) Condition 2; (C) Condition 3.

Kaplan–Meier curves indicated delayed conception during the post-FMD period across the study conditions (Figure 3). Heifers that did not conceive within the 180-day follow-up period were treated as right-censored observations. Cox proportional hazards analysis (Table 5) showed that, in Condition 1, the hazard of conception was significantly lower after FMD infection than before FMD infection (HR = 0.56; 95% CI: 0.46–0.67; p < 0.001). No significant baseline differences in conception hazard were detected between Condition 1 and Condition 2 (HR = 0.73; 95% CI: 0.49–1.10; p = 0.14) or Condition 3 (HR = 0.89; 95% CI: 0.72–1.09; p = 0.25). A significant interaction was observed between period and Condition 3 (HR = 1.38; 95% CI: 1.02–1.88; p = 0.04), indicating that the decline in the hazard of conception after FMD exposure was less pronounced in Condition 3 than in Condition 1. The interaction for Condition 2 was not significant (HR = 1.55; 95% CI: 0.88–2.72; p = 0.13). The proportional hazards assumption was met (global test, p = 0.49).

### Reproductive record exit

**Figure 4:**
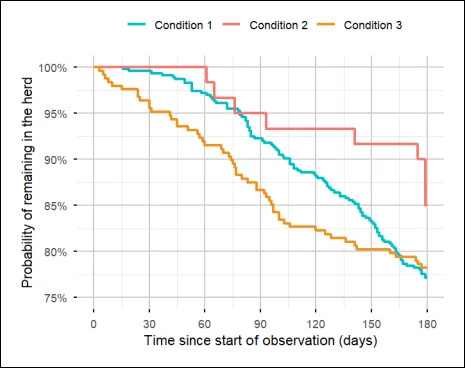
Unadjusted Kaplan–Meier survival curves showing time to reproductive record exit after foot-and-mouth disease (FMD) infection or vaccination in Holstein–Friesian heifers. Condition 1 = Heifers clinically affected by FMD during the outbreak; Condition 2 = Heifers developing clinical FMD after vaccination; Condition 3 = Clinically healthy heifers after vaccination.

Figure 4 shows the unadjusted Kaplan–Meier survival curves for time to reproductive record exit during the post-FMD period in HF heifers across the three study conditions. Although the curves partly overlapped, the pattern of herd retention changed over time, indicating non-constant differences in reproductive record exit hazard among conditions. Because the proportional hazards assumption was violated in the standard Cox model, reproductive record exit was further evaluated using a piecewise Cox proportional hazards model with follow-up divided into 0–90-day and 91–180-day intervals (Table 6).

Using Condition 1 as the reference group, no significant difference in reproductive record exit hazard was observed for Condition 2 during the first 90 days (HR = 0.73; 95% CI: 0.22–2.37; p = 0.60), whereas Condition 3 showed a significantly higher hazard than Condition 1 (HR = 1.97; 95% CI: 1.23–3.17; p < 0.01). During the later follow-up period (91–180 days), the time-varying effect remained non-significant for Condition 2 (HR = 0.64; 95% CI: 0.15–2.69; p = 0.54), whereas Condition 3 showed a significant reduction in hazard relative to its earlier period effect (HR = 0.32; 95% CI: 0.16–0.63; p < 0.01). Overall, the piecewise Cox model was significant (likelihood ratio test, p = 0.004), indicating that the association between exposure condition and reproductive record exit hazard varied over time.

**Table 5:** Cox proportional hazards model for FSCI.

**Variable**	**Coefficient**	**HR**	**95% CI**	**p-value**
FMD after	−0.59	0.55	0.46–0.66	<0.01
Condition 2	−0.31	0.73	0.49–1.10	0.13
Condition 3	−0.12	0.88	0.71–1.09	0.23
FMD after × Condition 2	0.44	1.56	0.89–2.73	0.12
FMD after × Condition 3	0.32	1.40	1.03–1.91	0.03

FMD = Foot-and-mouth disease; HR = Hazard ratio; CI = Confidence interval; FSCI = First-service-to-conception interval; n = 1544 observations; events = 730; concordance = 0.57; Wald test p < 0.001; global proportional hazards test p = 0.49. Cox proportional hazards regression was fitted with robust standard errors, clustered at the heifer level. The reference category was Condition 1 in the pre-exposure period. Thus, “FMD after” represents the post-exposure effect within Condition 1. “Condition 2” and “Condition 3” represent baseline differences compared with Condition 1 in the pre-exposure period. The interaction terms “FMD after × Condition 2” and “FMD after × Condition 3” represent how post-exposure changes in conception hazard differed in Conditions 2 and 3 relative to Condition 1. HR <1 indicates a lower hazard of conception and longer FSCI, whereas HR >1 indicates a higher hazard of conception and shorter FSCI.

**Table 6:** Piecewise Cox proportional hazards model for post-FMD reproductive record exit according to study condition in Holstein–Friesian heifers.

**Condition**	**Time interval (days)**	**n/N**	**Coefficient**	**HR**	**95% CI**	**p-valueᵃ**
2	<90	3/60	−0.32	0.73	0.22–2.37	0.60
	>90	6/60	−0.45	0.64	0.15–2.69	0.54
3	<90	33/248	0.68	1.97	1.23–3.17	<0.01
	>90	21/248	−1.14	0.32	0.16–0.63	<0.01

Condition 1 served as the reference group. Follow-up time was partitioned into 0–90-day and 91–180-day intervals. FMD = Foot-and-mouth disease; HR = Hazard ratio; CI = Confidence interval. HR >1 indicates an increased hazard of reproductive record exit relative to Condition 1, whereas HR <1 indicates a decreased hazard. ᵃ Overall model significance: Likelihood ratio test, p = 0.004. Statistical significance was set at p < 0.05.

## DISCUSSION

### Reproductive impact of FMD under field conditions

FMD is a highly contagious viral disease that spreads rapidly and remains a major challenge in many countries, including Indonesia, where the disease re-emerged in 2022 [35]. The incidence of FMD in Indonesia, particularly in Java during 2022–2023, reportedly reached 308,148 cases across multiple species, with 96.4% in cattle [36]. This situation contributed to reduced cattle populations, decreased milk production, increased livestock mortality [20], and higher rates of early embryonic loss [7]. Oktanella *et al.* [21] also reported decreased milk production following the outbreak. Consequently, mass vaccination has become the primary strategy for limiting transmission in susceptible cattle populations [37].

However, scientific evidence on the effects of FMD infection and vaccination on bovine fertility remains limited, although fertility is an important indicator of longer-term economic losses that may not be immediately apparent. Because reproductive performance is influenced by environmental and management factors, a robust evaluation requires before-and-after comparisons following exposure to the outbreak [38, 39]. In this study, a retrospective within-animal before-and-after approach was used to compare reproductive performance in HF heifers before-and-after natural FMD infection or vaccination. To the best of our knowledge, this is the first study to use this design to directly compare the reproductive consequences of natural FMD infection and vaccination in dairy heifers. It also represents one of the first detailed field-based assessments of reproductive performance following the 2022 re-emergence of FMD in Indonesia, after more than three decades free from the disease. Although this study was not designed to compare reproductive outcomes among different FMDV lineages, it provides field-based evidence of reproductive impairment following the 2022 Indonesian outbreak associated with the O/ME-SA/Ind-2001e lineage.

### Effects of natural FMD infection on fertility

Heifers directly infected with FMD (Condition 1) showed marked reductions in reproductive performance after exposure, reflected by significantly lower FSCR and PR. This decline was plausibly related to reduced feed intake during the early phase of infection, due to painful lesions on the lips and tongue [3]. Nutritional deficiency can impair ovarian and uterine function, thereby compromising embryo development and implantation [40]. In addition, the decline in PR may reflect the more persistent adverse effects of FMD in cattle than in other species, where these effects are often more transient [41].

In cattle, FMD infection has been associated with heat intolerance syndrome, respiratory distress, and reproductive disorders that may persist after apparent clinical recovery, with residual effects reported for years after major outbreaks [10, 42]. The reduced reproductive performance observed after natural FMD infection is unlikely to be attributable to generalized immunosuppression. Experimental studies have shown that cattle remain immunocompetent during the acute phase of FMDV infection, maintaining stable leukocyte counts and intact T-cell responses, with only minimal systemic cytokine dysregulation [43]. Therefore, reproductive impairment in Condition 1 is more plausibly explained by indirect physiological effects of infection, particularly disruption of energy metabolism, which may subsequently reduce fertility [44]. Although reproductive hormones were not directly measured in this study, prior evidence suggests that FMDV infection may disrupt reproductive endocrine function [45]. This decline in fertility after natural FMD infection is consistent with previous evidence from Indonesia, as Syah *et al.* [7] reported changes in AI success rates in HF cows following the FMD outbreak in the same province.

### Effects of FMD vaccination on fertility

Vaccination is a key strategy for protecting susceptible cattle populations against FMD transmission and mitigating the broader reproductive impacts of the disease. However, its effectiveness should also be evaluated in relation to reproductive performance [46]. Findings from Conditions 2 and 3 suggest that vaccination in heifers did not consistently produce marked adverse reproductive effects. In Condition 2, FSCR and PR did not differ significantly before-and-after vaccination, although PR was slightly lower after vaccination (p = 1.00 for FSCR and p = 0.57 for PR). In Condition 3, FSCR was not significantly altered (p = 0.72), whereas PR showed a modest but statistically significant decline after vaccination (p = 0.04). Importantly, the magnitude of this decline was smaller than that observed in heifers directly affected by natural FMD infection.

This difference is biologically plausible. Vaccination delivers a controlled dose of inactivated antigen [47], thereby limiting excessive immune activation and tissue damage [48, 49]. After vaccination with an inactivated FMDV vaccine, the immune system is primarily activated by the adjuvant and non-replicating viral antigens. This stimulation induces a controlled and localized innate immune response, resulting in mild systemic inflammation, such as transient fever and lethargy, and short-term physiological stress that typically resolves rapidly [50–52]. Unlike natural infection, vaccination induces a controlled immune response without viral replication, thereby avoiding the prolonged physiological stress associated with acute disease. This interpretation aligns with evidence that cattle do not experience immunosuppression during FMDV exposure [43], supporting the view that vaccination-related reproductive effects are transient and largely mediated by short-term inflammatory and metabolic adjustments rather than by immune dysfunction.

### Timing of AI after FMD infection or vaccination

In addition to the main before-and-after comparison, an exploratory analysis evaluated whether the interval between FMD infection or vaccination and the first post-exposure AI was associated with first-service conception outcomes. Table 3 showed a consistent pattern across all three conditions, in which first AI performed >90 days after FMD infection or vaccination was associated with the most favorable reproductive performance. Although a similar numerical trend was observed in Condition 2, the differences were not statistically significant, dulikely toe of the limited sample size and the sparse number of conception events in this group.

These findings are consistent with Garcia-Pintos *et al.* [53], who showed that FMD vaccination administered close to breeding or early pregnancy was associated with a higher likelihood of pregnancy failure. Together, these results suggest that the early post-infection or post-vaccination period may represent a vulnerable reproductive window, during which residual inflammatory, febrile, metabolic, or endocrine disturbances may compromise fertilization, early embryonic development, or pregnancy establishment.

### Conception interval and reproductive efficiency

The findings on the conception interval further support these interpretations. Heifers in Condition 1 showed delayed conception after exposure, indicating that the adverse reproductive effect of natural FMD extended beyond the first insemination and reduced overall reproductive efficiency during follow-up. Descriptively, S/C also tended to increase after exposure across all conditions, which was broadly consistent with the pattern of reduced fertility. However, because S/C was evaluated descriptively rather than inferentially, this finding should be interpreted cautiously.

### Reproductive record exit after FMD exposure

Reproductive record exit after FMD infection or vaccination was also assessed. Because all animals were survivors and follow-up focused on the post-exposure reproductive period, this outcome should be interpreted as a proxy measure of reproductive record exit rather than a confirmed culling endpoint attributable solely to reproductive failure. The highest proportion of removals during the 180-day observation period was observed among heifers that experienced clinical FMD during the outbreak (Condition 1). However, the between-condition comparison was more complex than suggested by crude proportions alone because the hazard of removal varied over time.

In particular, the removal pattern in Condition 3 was time-dependent, with distinct risk profiles in the early and late post-exposure periods. This finding indicates that herd removal after vaccination or outbreak exposure cannot be interpreted exclusively as a direct reflection of fertility status. Rather, it likely also reflects management decisions made under outbreak conditions, including concerns regarding infection risk, economic pressure, herd restructuring, or precautionary removal of animals perceived to be at higher epidemiological risk [54, 55].

### Overall implications

Overall, these results indicate that natural FMD infection had a substantially greater detrimental effect on reproductive performance in HF heifers than FMD vaccination, as reflected by clearer declines in conception outcomes and delayed pregnancy establishment. Although both naturally infected and vaccinated heifers showed reductions in FSCR and PR, these declines were 23% lower for FSCR and 26% lower for PR in vaccinated heifers. Given that FMD is among the most economically devastating livestock diseases, the overall benefits of vaccination for animal health, outbreak control, and economic protection likely outweigh the relatively limited reproductive effects observed after vaccination [53]. Therefore, vaccination programs remain essential for protecting cattle populations against infection and mitigating the broader reproductive consequences of FMD under field conditions.

### Limitations

Several limitations should be considered. First, the retrospective design relied on cooperative reproductive records, and several potentially important confounders, such as body weight, body condition score, semen batch quality, and AI technician identity, were not consistently available. Nutritional status, previous health history, farm-level management practices, and indicators of energy balance, including non-esterified fatty acids and β-hydroxybutyrate, were also inconsistently recorded and could not be fully adjusted for in the statistical analyses. Although the within-animal before-and-after design reduced some between-animal variability, residual confounding may have influenced the observed associations among FMD exposure, vaccination, and reproductive outcomes.

Second, reproductive record exit was based on a proxy definition because complete farm-level records of culling, sale, transfer, and mortality were unavailable. Therefore, this outcome should not be interpreted as confirmed culling because of infertility. Some heifers may have been sold, transferred to other farms, or lost from reproductive recording. Accordingly, reproductive record exit was interpreted as an exploratory outcome and was de-emphasized relative to the main fertility outcomes.

Third, the study was conducted within a single dairy cooperative, which may restrict the generalizability of the findings to other dairy production systems. However, the relatively large sample size (n = 772) and the use of real-world smallholder field data enhance the external validity of the results for comparable dairy production systems in Southeast Asia and other developing tropical regions.

## CONCLUSION

This retrospective within-animal before-and-after study demonstrated that natural FMD infection had a substantially greater detrimental effect on reproductive performance in HF heifers than FMD vaccination under smallholder dairy production systems in Indonesia. Heifers with clinical FMD during the outbreak exhibited significant reductions in FSCR and PR, together with delayed conception, indicating persistent impairment of reproductive efficiency following natural infection. In contrast, FMD vaccination was associated with relatively minor reproductive effects. Vaccinated heifers that remained clinically healthy showed only a modest reduction in PR, with no significant deterioration in FSCR. Furthermore, exploratory interval analyses suggested that delaying the first AI until more than 90 days after FMD infection or vaccination was associated with improved first-service conception outcomes, highlighting the importance of appropriate reproductive timing following disease exposure or immunization.

The principal strength of this study lies in its within-animal design, in which each heifer served as its own control, thereby minimizing confounding arising from individual genetic, physiological, and management differences. In addition, the large field-based dataset collected during the 2022 Indonesian FMD outbreak provides practical evidence directly applicable to smallholder dairy production systems. Nevertheless, interpretation of the findings should consider the retrospective nature of the study, the absence of several potentially important confounding variables, and the use of a proxy definition for reproductive record exit because complete culling records were unavailable.

From a practical perspective, the findings indicate that the reproductive consequences of natural FMD infection considerably outweigh the relatively limited effects associated with vaccination. These results support the continued implementation of FMD vaccination programs while emphasizing the importance of integrating vaccination schedules with reproductive management, particularly by avoiding insemination during the early post-vaccination or post-infection period whenever possible. Future prospective, multicenter studies incorporating endocrine, metabolic, and immunological biomarkers are warranted to clarify the biological mechanisms underlying FMD-associated reproductive impairment and to determine the optimal timing of breeding following infection or vaccination. Overall, maintaining effective vaccination programs together with appropriate reproductive management remains essential for minimizing fertility losses and improving the productivity and sustainability of dairy herds in FMD-endemic regions.

## DATA AVAILABILITY

The supplementary data can be made available from the corresponding author upon request.

## GENERATIVE AI DECLARATION

The authors declare that generative artificial intelligence (AI) tools were used solely to improve language, grammar, and readability during manuscript preparation. All scientific content, data analysis, interpretation of results, and conclusions were developed and verified by the authors. The authors take full responsibility for the accuracy, integrity, and originality of the work presented, and no AI tool was listed as an author. 

## AUTHORS’ CONTRIBUTIONS

HAS: Conceptualization, data collection, data analysis, and original draft preparation. WN: Study design and original draft preparation. PU and ADP: Data analysis and manuscript preparation. APAY: Conceptualization, study design, and manuscript review. NI, SW, MM, TES, SS, MR, KS, and TS: Supervision, data analysis, and critical revision of the manuscript. All authors have read and approved the final version of the manuscript.
